# Generation and Characterization of Yeast Two-Hybrid cDNA Libraries Derived From Two Distinct Mouse Pluripotent Cell Types

**DOI:** 10.1007/s12033-012-9561-4

**Published:** 2012-06-07

**Authors:** Ying Zheng, Xiaoying Tan, Joanna Pyczek, Jessica Nolte, D. V. Krishna Pantakani, Wolfgang Engel

**Affiliations:** 1 Institute of Human Genetics, University of Goettingen, Heinrich-Dueker-Weg 12, 37073 Goettingen, Germany; 2 Department of Histology and Embryology, Medical College of Yangzhou University, 11 Huaihai Road, Yangzhou, 225001 Jiangsu China

**Keywords:** Pluripotent stem cells, Protein–protein interactions, Yeast two-hybrid cDNA library, ESC, maGSC

## Abstract

Pluripotent stem cells have the therapeutic potential in future regenerative medicine applications. Therefore, it is highly important to understand the molecular mechanisms governing the pluripotency and differentiation potential of these cells. Our current knowledge of pluripotent cells is largely limited owing to the candidate gene/protein approach rather than studying the complex interactions of the proteins. Experimentally, yeast two-hybrid system (Y2H) is by far the most useful and widely used method to detect the protein–protein interactions in high-throughput screenings. Unfortunately, currently there is no GAL4-based pluripotent stem cell-specific cDNA library available for screening the interaction proteins impeding the large-scale studies. In this study, we report the construction of Y2H cDNA libraries derived from mouse pluripotent embryonic stem cells (ESCs) and multipotent adult germ-line stem cells (maGSCs) in GAL4-based Y2H vector system with very high transformation efficiency. Furthermore, we have constructed two different baits and screened for interaction partners in an effort to characterize the libraries and also as a part of our ongoing studies. Consequently, many putative interaction proteins were identified in both cases and their interaction was further validated by direct-Y2H. The observed interactions between bait proteins and their respective analyzed putative interaction proteins were further confirmed using two independent approaches in mammalian cells, thus highlighting the biological significance of the identified interactor (s). Finally, we would like to make these cDNA libraries as a resource that can be distributed to the research community.

## Background

Embryonic stem cells (ESCs) derived from inner cell mass of the pre-implantation stage embryos are pluripotent and have the competence to differentiate into all the germ layers including germ cells [1, 2]. Human ESCs hold the promise for future regenerative medicine therapies as they can self-renew without loosing the pluripotency and have the potential to differentiate into all the cell types of the body [3] albeit the ethical issues associated with ESCs derivation from embryo. On the other hand, multipotent adult germ-line stem cells (maGSCs) which are generated from adult mouse testis are as pluripotent as ESCs [4–8], and if successful, the human maGSCs hold great potential in regenerative medicine applications and can bypass the ethical issues associated with ESCs. However, it is important to fully understand how the pluripotency is established and maintained and how the differentiation is initiated and maturated to a desired cell-type(s) before we proceed with any clinical applications.

Previous efforts to understand the pluripotency of ESCs at molecular level have uncovered genes such as Oct3/4, Nanog, Sox2, Rex1, and Sall4 as important regulators of pluripotency [9–12]. However, our knowledge on pluripotency of ESCs is largely limited owing to the candidate gene/protein approach rather than studying the complex interactions of the protein, as the function of a specific protein may depend on its interacting protein. Therefore, studies aimed at understanding the protein–protein interaction networks (protein interactomes) of pluripotent cells have identified some important regulatory networks implicated in the pluripotency [13, 14]. Experimentally, protein–protein interactions can be detected using a variety of techniques such as yeast two-hybrid system (Y2H), immunoprecipitation-coupled mass spectrometry (IP-MS), protein microarrays, synthetic lethality, targeted releasable affinity probe (TRAP), stable isotope labeling by amino acids in cells (SILAC). Among the above mentioned, Y2H system allows the cost-effective and genomic-scale screening for protein–protein interactions in a relatively short period of time. This approach, which relies on the activation of downstream reporter genes by the GAL4-based system [15, 16], in which the transcriptional activator GAL4 is split into DNA-binding domain (BD) and activation domain (AD) and fused to bait and prey, respectively. The interaction between bait and prey proteins bring the GAL4 domains into close proximity to each other and lead to the transcriptional activation of reporter genes. Based on these properties, even weak and transient interactions, which are difficult to detect in immunoprecipitation based experiments can be detected easily.

Unfortunately, currently there is no pluripotent stem cell-specific cDNA library available for screening the interaction proteins impeding the large-scale studies. Here, we report the construction of high quality cDNA libraries of mouse ESCs and maGSCs in GAL4-based Y2H vector system. Furthermore, we have constructed two different baits [Zinc finger protein 819 (Zfp819) and Stimulated by retinoic acid (Stra8)] and screened for interaction partners in an effort to characterize the libraries and also as a part of our ongoing studies. Consequently, many interaction partners were identified in both cases and the putative interaction partners were validated by direct-Y2H. Further, one interaction partner from each screen was validated in mammalian cell culture system using co-immunoprecipitation (Co-IP) or glutathione *S*-transferase (GST) pull-down assay, and co-localization methods, highlighting the biological significance of our Y2H screen.

## Results and Discussion

### Generation of Mouse ESCs and maGSCs Y2H cDNA Libraries

Although many different techniques are available to analyze protein–protein interactions, only two techniques, IP-MS and Y2H screen are widely used in high-throughput screening applications. The use of IP-MS allows the identification of most of the components of a large protein complex, whereas Y2H identifies interactions between two individual proteins and their minimal interacting domains. For high-throughput protein interaction analyses, the Y2H screen is by far the most useful and widely used as it is based on the in vivo genetic screening approach and has the potential to identify low affinity as well as transient protein interactions [17]. In this study, we have successfully created Y2H cDNA libraries derived from mouse ESCs and maGSCs. ESCs, the “gold standard of pluripotency” are being studied extensively at the molecular level to understand the pluripotent cell characteristics and their potential in clinical applications. While maGSCs are a recently derived cell type and possess all the analyzed pluripotent cell characteristics [4–8], hence might be an alternative to ESCs to avoid ethical and immunological concerns.

**Fig. 1:**
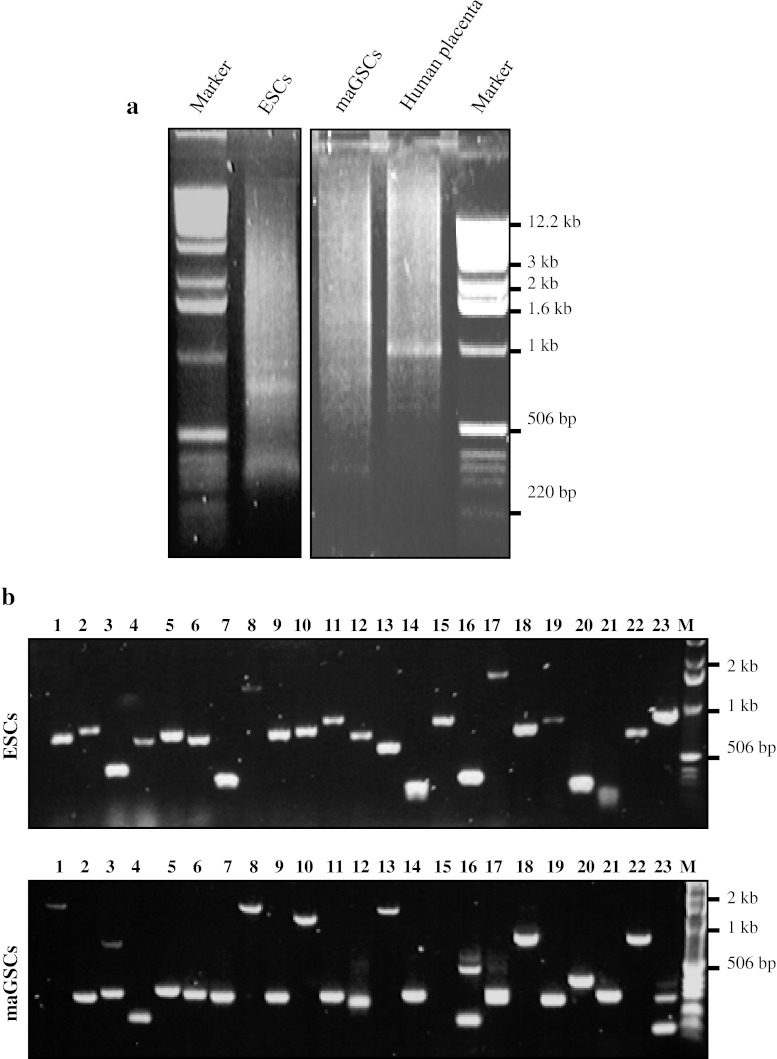
Construction and verification of Y2H cDNA libraries of mouse ESCs and maGSCs. **a** Agarose gel electrophoresis showing the smear of first strand cDNA synthesized from polyA+ mRNA isolated from mouse ESCs and maGSCs using oligo-(dT) priming method. The polyA+ mRNA from human placenta was used as a positive control. **b** Colony PCR amplification on randomly picked yeast recombinant clones from mouse ESCs and maGSCs Y2H cDNA libraries. The molecular weights of 1 kb DNA ladder are indicated in both **a** and **b**

Currently, there are no available cDNA libraries of mouse pluripotent cells for analyzing the protein–protein interactions in GAL4-based Y2H. To overcome this, we have created Y2H cDNA libraries from high quality polyA+ mRNA derived from mouse ESCs as well as from maGSCs using Oligo-(dT) priming method (Fig. 1). After first-strand cDNA synthesis, we obtained good smear from both ESCs and maGSCs which was comparable to the human placenta polyA+ mRNA, a positive control provided in the Matchmaker Library Construction and Screening Kits (Fig. 1a). Further, ds-cDNA (double strand-cDNA) was prepared using SMART technology and was cloned into pGADT7-Rec vector with the help of recombination in yeast AH109. The transformation efficiency was higher than the expected (≥1.0 × 10^6^) with ~2.3 × 10^6^ and ~2.4 × 10^6^ transformants for ESCs and maGSCs, respectively (Table 1). We then performed colony PCR on ~100 randomly picked transformants using vector specific primers to check the insert size and to analyze the recombination efficiency (Fig. 1b). The PCR analysis of the ESC cDNA library revealed the insert ranging from ~0.2 to ~2.0 kb with an average insert size of ~0.8 kb (Table 1). Similarly, maGSC library was found to contain inserts ranging from ~0.1 to ~2.0 kb with an average insert size of ~0.6 kb (Table 1). The observed average insert size of ~0.6 to ~0.8 kb of the prey clone will minimize the protein interaction domain. The percentage of positive recombinant clones was ~90 and ~94 % in ESC and maGSC libraries, respectively (Table 1). The remaining recombinant clones might have a longer insert size, hence could not be amplified. We then prepared frozen stocks of ESCs and maGSCs with a cell density of ~8 × 10^7^ and ~7 × 10^7^ cells/ml, respectively (Table 1). Furthermore, we estimated the titer of the libraries as ~4 × 10^7^ and ~3.6 × 10^7^ cfu/ml for ESCs and maGSCs libraries, respectively (Table 1).

**Table 1:** The mouse ESCs and maGSCs Y2H cDNA libraries transformation efficiency, insert size and, the quality of libraries

	ESCs cDNA library	maGSCs cDNA library
Transformation efficiency (expected ≥ 1 × 10^6^ transformants/3 μg pGADT7-Rec)	2.3 × 10^6^	2.4 × 10^6^
Insert size (kb)		
Minimum	0.2 kb	0.1 kb
Maximum	2.0 kb	2.0 kb
Average	0.8 kb	0.6 kb
% of positive recombinant clones	90	94
Cell density of frozen library (cells/ml)	8 × 10^7^	7 × 10^7^
cDNA library titer (cfu/ml)*	4 × 10^7^	3.6 × 10^7^

* cfu/ml – colony forming units/ml

### Characterization of Y2H cDNA Libraries

The quality of both ESC and maGSC libraries was evaluated by screening interaction proteins for Zfp819 and Stra8, respectively. *Zfp819* was identified in a comparative transcriptome analysis of undifferentiated and differentiated ESCs as a novel gene which is expressed highly in pluripotent cells but not in their differentiated counterparts ([6] and unpublished data). The protein encoded by *Zfp819* belongs to C2H2-zinc finger (C2H2-Znf) family of proteins and bears a functional KRAB (Krueppel-associated box) domain on its N-terminal region, yet the function is not known. Recently, *Zfp819* was shown to be highly expressed in partially and fully reprogrammed induced pluripotent cells (iPSCs), but not in parental somatic cells [18]. Collectively, these results suggest a possible crucial role for Zfp819 in establishment and maintenance of pluripotency. To elucidate the function of Zfp819 in pluripotent cells, we screened ESC cDNA library with N-terminal region of Zfp819 (Zfp819_N) as a bait. This screen yielded a total of ~800 colonies on high stringency nutritional selection plates (SD/-Leu/-Trp/-His/-Ade), of which 180 candidates were analyzed by sequencing, resulting in BLAST hits for 150 prey clones (Table 2). Further analysis revealed that 46 % of clones are in-frame of the target genes, of which 64 % of clones were identified as putative interaction proteins, whereas the remaining showed auto-activation in direct-Y2H assay. The putative interaction partners of Zfp819 were further categorized based on the GO (gene ontology) term biological process (Table 3) indicating that Zfp819 might function as a transcriptional and cell cycle/apoptosis regulator.

**Table 2:** Y2H screening of mouse ESCs and maGSCs cDNA libraries with bait proteins as a proof-of-concept

	No. of positive clones	Analyzed sequences	Sequences with BLAST hit	In-frame (%)	Out of-frame (%)	Minus (%)	3′ UTR (%)
ESCs library (Bait: Zfp819_N)	800	180	150	46.0	35.4	1.3	17.3
maGSCs library (Bait: Stra8^GA^)	300	81	76	26.0	37.0	0.0	37.0

**Table 3:** List of Zfp819 putative interaction proteins identified in a Y2H screen

Clone no.	NCBI accession No.	Gene/protein description	Gene ontology
174, 362	NM_145979	Chromodomain helicase DNA-binding protein 4 (Chd4)	Chromatin modification, regulation of transcription
270, 298, 313, 326, 372, 546, 548, 691	NM_008211	H3 histone, family 3B (H3f3b)	Nucleosome assembly
201, 532	NM_025828	Lectin, mannose-binding 2 (Lman2)	Protein transport
334, 354, 711, 720, 749	NM_008143	Guanine nucleotide binding protein (G protein), beta polypeptide 2 like 1 (Gnb2l 1)	Positive regulation of protein phosphorylation, negative regulation of translation
524	NM_010329	Podoplanin (Pdpn)	Cell morphogenesis
559	NM_012342	BMP and activin membrane-bound inhibitor, homolog (Bambi)	Positive regulation of cell proliferation, regulation of cell shape
314	NM_028388	NADH dehydrogenase (ubiquinone) flavoprotein 2 (Ndufv2)	Mitochondrial electron transport, NADH to ubiquinone
315	NM_023202	NADH dehydrogenase (ubiquinone) 1 alpha subcomplex, 7 (Ndufa7)	Mitochondrial electron transport, NADH to ubiquinone
318	NM_027204	Mitochondrial ribosomal protein L12 (Mrpl12)	Transcription from mitochondrail promoter, translation
330	NM_011157	Serglycin (Srgn)	Apoptosis, platelet degradation
194	NM_026274	Ring finger and SPRY domain containing 1 (Rspry1)	NA
196, 285, 265, 264, 236, 252, 271, 276, 700, 729	NM_010860	Myosin, light polypeptide 6, alkali, smooth muscle and non-muscle (Myl6)	Skeletal muscle tissue development, muscle contraction
356	NM_013535	Gene rich cluster, C10 gene (Grcc10)	NA
267, 302, 360	NM_001143790	RIKEN cDNA 1500010J02 gene	Positive regulation of DNA replication, telomere maintenance
561	NM_025849	RIKEN cDNA 3110001D03 gene	NA
368, 550, 568	NM_026566	RIKEN cDNA9430023L20 gene	Autophagy
286, 248	NR_015585	RIKEN cDNA 4933439C10 gene	NA

**Fig. 2:**
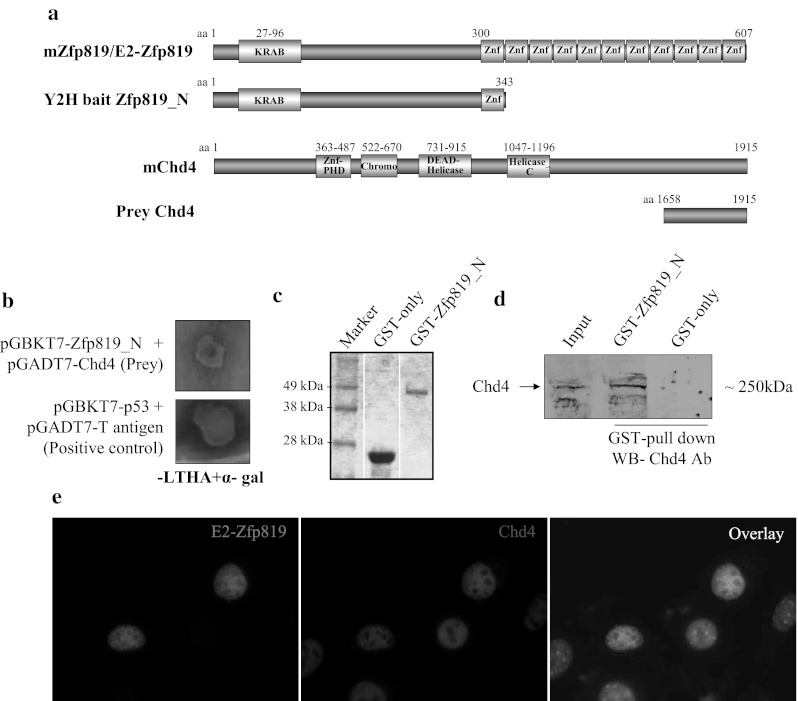
Zfp819 interacts with Chd4 in both ex vivo and in vivo. **a** Illustration of mouseZfp819 and Chd4 proteins together with Y2H/mammalian expression constructs used for the analysis. Zfp819 contains a kruppel-associated box (KRAB) domain at the N-terminus and 11 tandemly arranged zinc finger (Znf) motifs at the C-terminus of the protein. The Chd4 contains a Znf-plant homeodomain (Znf-PHD), a chromatin organization modifier (Chromo) domain and two centrally located helicase domain types, DEAD-helicase (DEAD) and helicase carboxyl-terminal domain (helicase_C). **b** Confirmation of Chd4 interaction with Zfp819 using direct-Y2H. The AH109 yeast cells transformed with bait and prey plasmids, as indicated, were selected on nutritional selection medium, SD-LTHA and for α-galactosidase (α-gal) activity. As a positive control, the interaction between p53 and T-antigen was assayed in direct-Y2H experiments. **c** Coomassie blue stained SDS-PAGE gel showing the homogeneity of purified GST-only or GST-Zfp819_N fusion proteins. **d** GST pull-down assay and subsequent Western blot analysis with Chd4 antibodies confirmed the interaction between Zfp819 and endogenous Chd4. **e** IFC analysis of NIH-3T3 cells transiently transfected with E2-Zfp819 (*green*) showed co-localization of Zfp819 with endogenous Chd4 (*red*) in the nucleus. The cells were counterstained with DAPI to visualize the nucleus (Color figure online)

In order to validate the authenticity of our Y2H screen, we performed protein interaction studies between Zfp819 and its putative interaction partner, Chromodomain helicase DNA-binding protein 4 (Chd4), using direct-Y2H and mammalian cell culture system (Fig. 2). Chd4 is a component of NuRD chromatin remodeling complex, which functions in translational repression by histone deacetylation [19]. We have identified the C-terminal region (aa 1,658–1,915) of Chd4 as a prey in our Y2H screen, narrowing down the Chd4 interaction region with Zfp819 (Fig. 2a). Co-transformation of purified prey Chd4 clone and bait, Zfp819, confirmed the interaction between Chd4 and Zfp819 in a direct-Y2H assay and showed no auto-activation when Chd4 was co-transformed together with empty bait vector (Fig. 2b and data not shown). To perform GST pull-down assay, we purified the GST-Zfp819_N fusion protein to the near homogeneity (Fig. 2c). We also purified GST protein alone to use as a negative control (Fig. 2c). The GST pull-down assay with cell extracts from ESCs and the subsequent western blot analysis with Chd4 specific antibodies confirmed the interaction between Zfp819 and Chd4 (Fig. 2d). Co-localization studies with E2-Zfp819 and endogenous Chd4 revealed that both proteins partially co-localize in the nucleus as discrete spots (Fig. 2e).

**Fig. 3:**
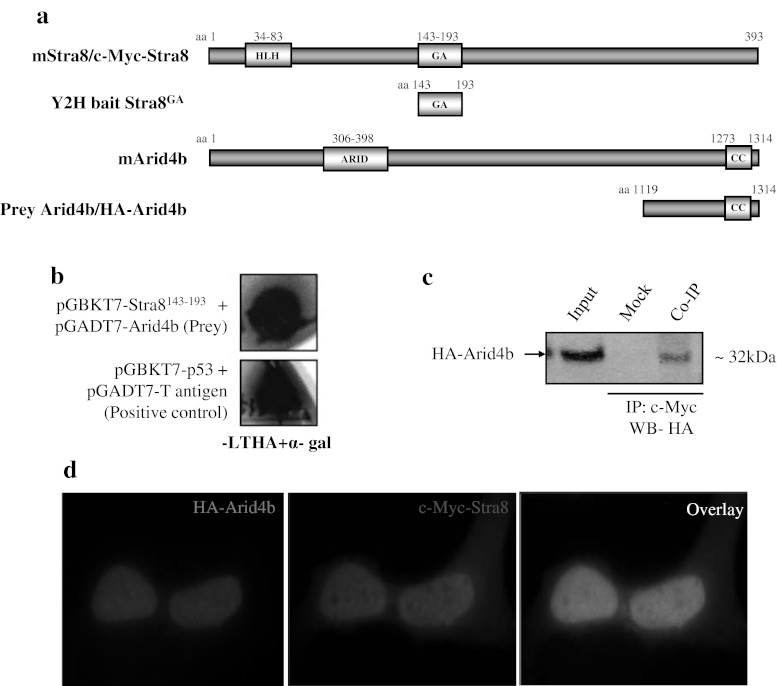
Stra8 interacts with Arid4b in both ex vivo and in vivo. **a** Illustration of mouseStra8 and Arid4b protein domain organization together with Y2H/mammalian expression constructs used for the analysis. Stra8 contains an N-terminal helix–loop–helix (HLH) domain and a centrally located GA-rich region. Arid4b bears an AT-rich interaction domain (ARID) and C-terminally located coiled-coil domain. **b** Confirmation of Arid4b interaction with Stra8 using direct-Y2H. The yeast cells transformed with bait and prey plasmids, as indicated, were selected on SD–LTHA and for α-galactosidase (α-gal) activity. The interaction between p53 and T-antigen was assayed as a positive control in direct-Y2H experiments. **c** Co-IP analysis using c-Myc antibodies on cell lysates prepared form NIH-3T3 cells transiently transfected with c-Myc-Stra8 and HA-Arid4b. Western blot analysis with HA-tag antibody confirmed the interaction between Stra8 and Arid4b. **d** IFC analysis of NIH-3T3 cells transiently transfected with c-Myc-Stra8 (*green*) and HA-Arid4b (*red*) showed co-localization of both proteins in the nucleus. The cells were counterstained with DAPI to visualize the nucleus (Color figure online)

On the other hand, we used Stra8 as bait to screen maGSCs library. Stra8 is a retinoic acid responsive gene essential for meiosis, but is also known to be expressed in pluripotent cells, yet the molecular function is unknown [20–24]. Screening of maGSCs cDNA library with glutamic acid (GA)-rich region of Stra8 (Stra8^GA^) resulted in a total of ~300 colonies on high stringency nutritional selection plates. Out of 300 positive clones, 81 were analyzed by sequencing, resulting in a BLAST hits for 76 prey clones (Table 2). In contrast to ESCs screen, maGSCs screen resulted in 26 % of in-frame clones; while 37 % clones contained 3′ UTR sequences (Table 2). The direct-Y2H analysis of in-frame clones identified 40 % as putative interaction proteins, while the remaining 60 % showed auto-activation. The GO analysis of Stra8 putative interaction partners revealed that Stra8 might function in chromatin assembly/modification and transcription regulation processes (Table 4). To validate the Stra8 Y2H screen, we characterized the interaction between Stra8 and its putative interaction partner, AT-rich interactive domain 4B (Arid4B), in both ex vivo and in vitro studies (Fig. 3). The C-terminal region (aa 1,119–1,314) consisting of coiled–coiled (CC) domain of Arid4B has been identified as a prey, indicating that this region is sufficient to mediate the interaction with Stra8 (Fig. 3a). The direct-Y2H assay further confirmed the interaction of Arid4B with Stra8 and showed no auto-activation (Fig. 3b and data not shown). Transient overexpression of c-Myc-tagged Stra8 (c-Myc-Stra8) and HA-tagged Arid4B (HA-Arid4B), and subsequent Co-IP studies revealed the interaction between these two proteins (Fig. 3c). Further, overexpression and immunostaining studies revealed that both Stra8 and Arid4B co-localize in the nucleus with diffused pattern (Fig. 3d).

**Table 4:** List of Stra8 putative interaction proteins identified in a Y2H screen

Clone no.	NCBI accession no.	Gene/protein description	Gene ontology
62	NM_194262	AT-rich interactive domain 4B (Arid4B)	Chromatin assembly, regulation of transcription
50	NM_030241	SET domain containing (lysine methytransferase) 8 (Setd8)	Chromatin modification, negative regulation of transcription
81, 169	NM_001167922	General transcription factor II E, polypeptide 2 (Gtf2e2)	Regulation of transcription, transcription elongation
52, 166	NM_010480	Heat shock protein 90, alpha (cytosolic), class A member1 (Hsp90AA1)	Mitotic cell cycle, protein folding, ATP catabolic process
108	NM_009288	Serine/threonine kinase 10 (STK10)	Protein phosphorylation
231	NM_026396	Biogenesis of ribosome, homolog (S. cerevisiae) (Brix1)	Translation, ribosome biogenesis

Generally, Y2H screen is prone to result in detection of false positives, albeit at low rate, hence the relevance of the identified putative interaction proteins in a physiological context has to be validated using one or more independent interaction methods [25]. The confirmation of Chd4 and Arid4b interaction with Zfp819 and Stra8, respectively, strongly suggests that the other identified interaction partners of our screens are potentially to be biologically significant, but have to be verified using independent methods. Previously, several studies have successfully used Y2H screening method to detect physiologically relevant interaction partners for DNA-binding proteins, including zinc finger proteins [26–29]. The study by Kalenik et al. [27] has successfully used zinc finger protein YY1, which is implicated in the negative regulation of myogenic differentiation, in Y2H screen and identified interaction proteins such as YY1-associated factor 2 (YAF2). The interaction between YY1 and YAF2 was confirmed through several independent methods employing mammalian cell culture system and further studies revealed that YAF2 binds to YY1 and enhances the proteolytic cleavage of this factor during myogenic differentiation [27]. Likewise, Rodel et al. [28] have used Gfi-1, a zinc finger protein with a dominant oncogene function, to screen for interaction proteins using Y2H assay and could identify PIAS3, an inhibitor of STAT signaling, as an interaction partner. The authors could confirm the physical interaction between Gfi-1 and PIAS3 through various independent experimental methods and show that Gfi-1 is a novel component of STAT signaling pathway with a function in relieving PIAS3 block and activation of STAT signaling [28]. Moreover, two other zinc finger proteins, INSM1 and A20 were successfully used in Y2H screens to identify physiologically relevant interaction proteins such as CAP and TXBP151, respectively [26, 29]. Collectively, all of these findings together with results from our current Y2H screen point to the identification of biologically significant interaction proteins in Y2H screening approach.

## Conclusions

In conclusion, we have generated high quality Y2H cDNA libraries from two distinct mouse pluripotent cell types, ESCs and maGSCs. Further, as a proof of concept, we have performed Y2H screen on these two libraries with two different bait proteins and could identify several putative interaction partners, thus validating the quality of these libraries. We also validated the interaction between bait proteins and their respective putative interaction protein using in vivo, ex vivo, and in vitro protein–protein interaction methods, thus highlighting the biological significance of the identified interactor. Finally, our Y2H cDNA libraries are useful tools to analyze the protein–protein interactions in pluripotent stem cells and will be distributed freely for the research community.

## Methods

### Cell Culture

The derivation and maintenance of mouse ESCs and maGSCs from 129/Sv genetic background was described previously [7]. Briefly, the undifferentiated ESCs and maGSCs were maintained on mitomycin C-inactivated mouse embryonic fibroblasts (MEFs) and cultured in DMEM (PAN, Germany) supplemented with 20 % defined FBS (fetal bovine serum) (PAN, Germany), 1 % penicillin/streptomycin, 0.1 mM non-essential amino acids, 2 mM l-glutamine, 1 mM sodium pyruvate, 0.1 mM β-mercaptoethanol (all the above ingredients are from Life Technologies, Germany) and 1,000 U/ml LIF (Chemicon, USA). For feeder depletion, the ESC and maGSC cultures were trypsinized and replated on gelatine coated culture dishes for 20 min. The resulting non-adherent ESCs and maGSCs were collected and used for further analysis. NIH-3T3 cells were maintained as previously described [30].

### Construction of GAL4-AD Fusion cDNA Libraries of ESCs and maGSCs

The GAL4-AD fusion cDNA libraries of ESCs and maGSCs were generated using Matchmaker Library Construction and Screening kits (Clontech, Germany) and the protocols provided therein. Briefly, total RNA was isolated from feeder depleted ESCs and maGSCs using peqGOLD TriFast-Reagent (PeqLab, Germany). Then, 1 mg of total RNA from each cell type was used to isolate polyA+ mRNA using Oligotex Direct mRNA kit (Clontech, Germany). Further, the polyA+ mRNA was used to synthesize first-strand cDNA using SMART cDNA synthesis technology (Clontech, Germany). To prepare sufficient double strand-cDNA (ds-cDNA) for transformation into yeast, first-strand cDNA was PCR amplified using Advantage 2 PCR kit (Clontech, Germany). The purified ds-cDNA was co-transformed with linear pGADT7-Rec vector into Yeast AH109 using lithium acetate transformation method [31] and selected on SD/-Leu agar plates. The resultant transformants were pooled and stored at −80°C after estimating the transformation efficiency. Also, cDNA inserts were PCR amplified from randomly picked colonies using Advantage 2 PCR kit (Clontech, Germany) to analyze the average length of cDNA inserts and the recombination efficiency.

### Y2H Screening using cDNA Libraries of ESCs and maGSCs, Respectively

To test the GAL4-AD fusion cDNA libraries of ESCs and maGSCs in Y2H screen, two bait proteins, Zfp819 and Stra8 were used, respectively. The GAL4-BD fusion bait construct of *Zfp819* was prepared by cloning PCR fragments of N-terminus Zfp819 (aa 1–343 corresponding to nucleotides (nt) 228–1,256 of NM_028913.3) into pGBKT7 vector resulting in pGBKT7-Zfp819_N. To prepare Stra8 bait construct (pGBKT7-Stra8^GA^), the cDNA fragment spanning the GA-rich region of the mouse *Stra8* gene (aa 143–193 corresponding to nucleotides (nt) 528–680 of NM_009292.1) was PCR amplified and cloned into the pGBKT7 vector containing the GAL4 DNA-binding domain. The bait constructs were co-transformed with empty pGADT7 vector into AH109 yeast strain to test and exclude auto-activation of GAL4 activated reporter genes, HIS3, ADE, and LacZ. After verification, the bait constructs were transformed separately into Y187 yeast strain. The Y2H screen was performed on ESCs and maGSCs cDNA libraries using pGBKT7-Zfp819_N and pGBKT7-Stra8^GA^, respectively, using Matchmaker pre-transformed library protocol (Clontech). Briefly, the pre-transformed ESCs and maGSCs libraries in yeast strain AH109 were mixed and mated together with strain Y187 containing the pGBKT7-Zfp819_N and pGBKT7-Stra8^GA^, respectively. After 24 h of mating, the culture was spread on SD/-Leu/-Trp/-His/-Ade plates and the surviving colonies were further verified on SD/-Leu/-Trp/-His/-Ade/+ X-α-Gal. The positive clones that were blue on X-α-Gal were cultured and the plasmid DNA was isolated using QIAprep Spin Miniprep Kit (Qiagen, Germany). The cDNA inserts of the isolated prey clones were PCR amplified and sequenced using vector specific primers. Identities of prey cDNA clones were determined by BLAST analysis (http://blast.ncbi.nlm.nih.gov/Blast.cgi).

### Direct-Y2H Assay

The prey cDNA clones were co-transformed with either empty pGBKT7 vector or bait construct into AH109 strain by lithium acetate method [31], to test the auto-activation or interaction, respectively. The co-transformants were first selected on SD/-Leu/-Trp plates and later tested for the reporter gene expression on SD/-Leu/-Trp/-His/-Ade and X-α-Gal plates.

### Construction of Mammalian Expression Vectors

To generate Zfp819-E2 expression construct, firstly, the CMV promoter of pEGFP-N1 (Clontech) was replaced with the human EF1α promoter to obtain phEF1α-EGFP-N1. Next, the ORF of mouse *Zfp819* was PCR amplified using primers containing the E2-tag at the C-terminus and cloned downstream of the human EF1α promoter in phEF1α-EGFP-N1 by replacing EGFP cassette to generate phEF1α-Zfp819-E2 construct. To generate c-Myc-tagged Stra8 expression vector, the ORF of the full-length mouse *Stra8* was PCR amplified and cloned into pCMV-Myc vector (Clontech). The cDNA insert of prey clone, *Arid4b* (aa 81–286 corresponding to nt 244–861 of NM_030241) was cloned into the pCMV-HA expression vector (Clontech).

### Purification of GST-Fusion Protein and GST Pull-Down Assay

For generation of GST-Zfp819_N fusion protein, the N-terminal region of Zfp819 (aa 1–343 corresponding to nucleotides (nt) 228–1,256 of NM_028913.3) was PCR amplified using pGBKT7-Zpf819_N construct as a template, and cloned into *Nco* I and *Not* I restriction sites of pET-41a vector (Novagen). The primers used for the PCR amplification is as follows: forward primer: 5′ CCATGGAGATGGCTGCTGACATGAATTTC and reverse primer: 5′ GCGGCCGCCAGGCTGGATGTACTGGGAAG. The expression and purification of either GST-only or GST-Zfp819_N fusion protein was performed as previously described [32]. For GST pull-down experiments, the ESCs from a confluent 10-cm cell culture plate were lysed with 1 ml of RIPA buffer and are processed as described [32].

### Co-IP, Western Blot, and Co-localization Studies

For Co-IP experiments, NIH-3T3 cells were transiently transfected with indicated constructs, using Lipofectamine 2000 (Life technologies, Germany) and processed using the Immunoprecipitation kit (Protein G) (Roche, Germany). The protein complexes were immunoprecipitated using c-Myc tag (05-724, Millipore) antibodies and western blotting was performed with HA-tag (ab9110, Abcam) antibodies. The eluted protein complexes from GST pull-down experiments were subjected to Western blotting using Chd4 antibodies (ab70469). For IFC experiments, NIH-3T3 cells grown on round coverslips were transiently transfected with indicated constructs. After 24 h, the cells were washed with PBS and fixed in 4 % paraformaldehyde (PFA) before processing for IFC using a standard protocol using the antibodies mentioned above. Finally, the coverslips were mounted with DAPI mounting medium (Vector Laboratories) and visualized by Olympus BX60 fluorescence microscope. Images were acquired and processed using Cell^F software.
